# The perception of patient safety in an alternate site of care for elective surgery during the first wave of the novel coronavirus pandemic in the United Kingdom: a survey of 158 patients

**DOI:** 10.1186/s13037-021-00284-8

**Published:** 2021-03-12

**Authors:** George Lee, Oliver T. Clough, Joseph A. Walker, Raymond E. Anakwe

**Affiliations:** 1 Department of Trauma & Orthopaedic Surgery, St Mary’s Hospital, Imperial College Healthcare NHS Trust, Praed Street, London, UK; 2 Department of Surgery, Imperial College, South Kensington, London, UK

**Keywords:** COVID-19, Elective care, Patient experience, Independent sector, Patient safety, Planned procedures

## Abstract

**Background:**

We undertook a prospective qualitative survey to ascertain the perceptions and experience of National Health Service patients in the United Kingdom who underwent planned or elective procedures and surgery at alternate ‘clean’ hospital sites during the coronavirus disease 2019 (COVID-19) pandemic. These alternate ‘clean’ hospital sites were independent hospitals running active staff and patient testing programmes for COVID-19 and which did not admit or treat patients suffering with COVID-19.

**Methods:**

A prospective survey was undertaken to include patients at least 30 days after a planned surgery or procedure conducted at a ‘clean’ alternate hospital site during the COVID-19 pandemic. The study was conducted using structured interviews undertaken by trained assessors. A 20% sample group of patients were randomly selected to participate in this study. Qualitative data related to confidence, safety and perceptions of safety were collected.

**Results:**

Ninety-five patients (60%) reported that they had prior worries or concerns about undergoing an elective procedure during the COVID-19 pandemic. A total of 47 patients (30%) had delayed their surgery at least once because of these concerns. A total of 150 patients (95%) felt that the precautions in place to protect their safety in the setting of an alternate ‘clean’ hospital site were well thought out and proportionate. Patients reported high levels of confidence in the measures undertaken. Separation of patient pathways using the independent sector and patient testing were identified by patients as having the greatest impact on their perception of safety.

**Conclusions:**

Patient confidence will be key to ensuring uptake of planned and elective procedures and surgery during the COVID-19 pandemic. Perceptions of safety will be key to this confidence and efforts to demonstrably enhance safety are well received by patients. In particular, patients felt that a dedicated programme of patient testing and separation of patient pathways provided the greatest levels of confidence in the safety of their treatment.

## Introduction

Patient perception of safety has been shown to impact on their likelihood to accept treatment, levels of patient satisfaction and on final patient treatment outcomes [1]. The global COVID-19 pandemic has resulted in the suspension of programmes of planned investigation and treatment for patients worldwide [2]. This includes cancer care as well as urgent cardiac, orthopaedic and gynaecologic surgical and diagnostic procedures. This was to free-up inpatient and critical care beds, maximise staff availability, and prepare for the anticipated large numbers of patients requiring respiratory support. In addition, the highly infectious nature of the severe acute respiratory syndrome coronavirus 2 (SARS CoV-2) coronavirus might expose patients to an unacceptable infectious risk. This worldwide suspension of planned and elective treatment has left millions of patients suffering the physical and mental effects of delayed treatment [3–7]. The resurgence of the pandemic with ‘second’ and ‘third’ waves presents a challenge as to how routine and urgent non-COVID related care can be safely provided. There has been a growing realization that the pandemic may be prolonged over an extended period and elective planned services will need to be delivered in this environment with measures to mitigate and stratify risks [8].

Widespread reports indicate that even when services have been re-established, patients have been reluctant, and afraid to take up scheduled appointments [9–11]. Hospitals have put a number of mitigations in place: COVID-19 testing programmes for patients and staff; separating emergency and planned elective patient cohorts from each other; enhanced cleaning programmes; restricting access to hospital sites including access for patient visitors; requiring patients to self-isolate for up to 14 days before a procedure or surgery.

Perhaps the most extreme example of patient cohort separation has been the co-operation between the National Health Service (NHS) and independent private healthcare providers in March 2020 at the height of the pandemic. This meant that planned elective care for public NHS patients which had been suspended could be delivered in ‘COVID-free’ non-acute private hospitals across the country, completely separate from hospitals providing care for patients infected with SARS CoV-2.

We undertook a study to ascertain the perceptions and experience of NHS patients who underwent elective procedures or surgery at alternate ‘clean’ sites during the COVID-19 pandemic.

## Methods

### Hypothesis

We hypothesized that patients would be confident in the precautions and safeguards put in place to enhance their safety while undergoing elective surgical procedures during the COVID-19 pandemic. In particular, we hypothesized that a dedicated programme of COVID testing for patients and staff as well as physical separation of patient pathways by using an alternate ‘clean’ hospital site not providing acute care for COVID-19 patients, would be the main contributors to patient confidence and perception of their safety.

### Study design

The study was designed as a qualitative study using data from interviews with patients who underwent elective planned surgery or procedures at an alternate ‘clean’ hospital site between 23 March 2020 and 7 September 2020 during the first wave of the COVID-19 pandemic in the United Kingdom. The data were collected through individual semi-structured interviews with 158 patients.

### Patients

We reviewed the electronic patient records at a major NHS acute hospital provider in an urban setting for all patients who had a planned elective procedure or surgery transferred to be performed at a ‘clean’ alternate hospital site under these arrangements between 23 March 2020 and 7 September 2020, the first wave of the pandemic in the United Kingdom.

We selected patients who had undergone an elective surgical procedure in this time frame and performed in this ‘clean’ environment and undertook a simple prospective patient survey at a minimum of 30 days following surgery to establish and review the perspectives and experience of these patients. Patients were selected if they had undergone a planned elective surgical procedure during the study period at an alternate ‘clean’ hospital site where patients with COVID-19 were not being admitted or treated. Five independent hospitals were used as alternate ‘clean’ hospital sites for elective surgical care. All hospitals provided a regular programme of polymerase chain reaction (PCR) COVID-19 swab testing for patients and staff. All hospitals also deployed an enhanced cleaning programme and restricted non-essential footfall on site so that no routine visitors were permitted.

Patients followed a defined pathway where once selected for surgery, they were required to self-isolate for a minimum of 72 h and up to 14 days before their procedure. All patients were required to practice meticulous physical social distancing and hand hygiene for the 14 days prior to their surgery they all had a negative PCR COVID swab test at least 72 h before the planned surgical procedure. Testing was provided in real time with testing and reporting of results within 24-h.

We identified 1150 patients and of these, we randomly selected a sample of 20% to form the patient study group. Two hundred and thirty randomized patients were identified. Three trained interviewers telephoned each patient to undertake a structured telephone interview. Patients described their perceptions and experience of their treatment during the COVID pandemic. They numerically rated their perception of safety during their elective admission, on a scale from 0 to 10, with 0 representing ‘feeling very unsafe’ and 10 indicating ‘feeling very safe’. We asked patients to rank the factors contributing to their confidence in the safety of their treatment. All interviews were conducted over a three-week study window and patients who did not respond initially were followed up with 2 further telephone calls.

### Structured interview

Structured interviews were undertaken by telephone by one of 3 trained interviewers. Patients were invited to respond to seven key questions answering from pre-determined choices. Interviewers were also able to capture qualitative data in the form of individual free-text additional comments.

Patients were asked:

- 1. Please rank the following in order of importance for you with respect to your safety during your recent surgery/procedure and reducing the risk of contracting COVID-19
  Patient testing for COVID-19; Staff testing for COVID-19; Enhanced cleaning; Restrictions on visitors; undergoing surgery in an alternate ‘clean’ site hospital where patients with COVID-19 are not admitted
- 2. Did you feel that the precautions taken to keep you safe from contracting COVID-19 during your recent surgery were effective, reasonable and proportionate?
  Yes / No.
- 3a. Have you had any concerns about undergoing a planned or elective surgery during the COVID-19 pandemic?
  Yes / No.
- 3b. Have you delayed undergoing this surgery or procedure because of worries or concerns about contracting COVID-19?
  Yes / No.
- 4. Would you support the continued use of alternative hospital sites in the independent sector for planned elective surgery during the COVID-19 pandemic funded by the health service?
  Yes / No.
- 5a. If the same service and separation of patients could be achieved in a public or state hospital would you be happy to have your treatment there?
  Yes / No.
- 5b. Would you be prepared to travel for up to an hour to access planned / elective treatment in a hospital that does not admit or treat patients with COVID-19?
  Yes / No.
- 6. Please score the level of confidence that you had in the measures taken to protect you from developing covid-19 during your recent admission and how safe you felt using a a scale from 0 to 10, with 0 representing ‘feeling very unsafe’ and 10 indicating ‘feeling very safe’
- 7. Is there anything else you would like to share with us about your perceptions of safety and experience during your recent surgery?

Patient assessment of their safety during the episode of care was the primary outcome measure. This was expressed as a numerical score (0–10). Secondary outcome measures were patient ranking of the safety measures in place, qualitative patient perceptions of their safety and the prevalence of hospital acquired COVID-19 infection in this patient group.

### Statistical analysis

Data were analysed using IBM SPSS® Statistics Version 26.0. Simple descriptive analysis only was required. Numerical scores were expressed as means with ranges.

## Results

We identified 1150 patients undergoing a procedure or surgery at a ‘clean’ alternate hospital site during this period (Table 1). Of the 230 patients forming the study group, 3 patients were excluded because a language barrier meant that they were unable to participate. 227 telephone interviews were attempted and 158 (70%) patients agreed to participate. Patients were interviewed after a mean of 42 (range, 33–64) days following their surgery.

**Table 1:** Specialty cases undertaken in a ‘clean’ alternate independent hospital

	Total Cohort	Share	Sample Cohort	Share
**Gynaecology/Gynae Onc**	318	27·7%	62	26·9%
**Urology**	213	18·5%	37	16·1%
**Cardiology**	132	11·5%	24	10·4%
**Trauma & Orthopaedics**	111	9·7%	31	13·5%
**General Surgery**	85	7·4%	13	5·7%
**Gastroenterology**	68	5·9%	14	6·1%
**Cardiothoracic**	48	4·2%	9	3·9%
**Ophthalmology**	36	3·1%	7	3·0%
**Colorectal**	27	2·3%	5	2·2%
**Nephrology**	25	2·2%	9	3·9%
**Breast**	24	2·1%	7	3·0%
**Vascular**	22	1·9%	3	1·3%
**Plastics**	21	1·8%	4	1·7%
**ENT**	13	1·1%	4	1·7%
**Hepatobiliary/Pancreatic**	5	0·4%	0	0·0%

Ninety-five patients (60%) reported that they were afraid or had serious concerns about attending hospital during the COVID-19 pandemic. Forty-seven patients (30%) had delayed their procedure or operation at least once for this reason. Of those who reported that they had delayed treatment because of their concerns, 35 patients (74%) accepted their procedure or operation date because this was offered at a ‘clean’ independent hospital site. However, considering the full patient group, 111 patients (70%) reported that they would have accepted the same procedure or surgery at an acute hospital on an NHS site if the same pathway separation could be guaranteed.

One hundred and fifty patients (95%) thought that the precautions put in place for their elective treatment in the alternate ‘clean’ hospital site were well thought out and proportionate. Patients assessed the level of safety during their admission very favorably with a mean score of 9 ∙ 2/10 (range, 5–10) on the numerical rating scale. A total of 150 patients (95%) were separated from other patients and accommodated in a single room during their admission. Patient visitors were not allowed in any cases.

Patients perceived being treated at a ‘clean’ site as the most important factor contributing to their safety. 73 of 158 (46%) patients ranked this factor first while 32 (20%) patients cited patient testing for COVID-19, 25 (16%) of patients cited having an individual room and 15 (9.5%) patients cited enhanced environmental cleaning. Staff testing for COVID-19, pre-procedure patient isolation and restrictions on patient visitors were perceived as less important contributors to patient safety (Table 2). All patients remained free of COVID-19 symptoms during their admission and up until the time of their telephone interview which was at least 30 days following the procedure.

**Table 2:** Patient ranking of factors contributing to their perception of safety

*Factors*	1st	2nd	3rd	Percentage Score
**Separation of pathway. Move elective care to ‘clean’ independent site**	73	31	14	31·1%
**Patient testing for COVID-19**	32	29	34	19·8%
**Separate/Individual Room (separation)**	25	48	26	20·8%
**Enhanced cleaning of hospital**	15	10	17	8·6%
**Pre-procedure self-isolation (14 days)**	7	6	9	4·4%
**Restriction on hospital visitors**	2	3	2	1·5%
**Staff Testing for COVID-19**	2	2	4	1·5%
**Not applicable/Blank**	2	29	52	12·2%

A total of 153 patients (97%) reported that they supported the paid arrangement between the NHS and the independent sector to provide separate ‘clean’ sites for elective procedures and surgery. 133 patients (84%) did say that they would be willing to travel up to an hour to be treated at a ‘clean’ site within the public sector or NHS should these be established.

## Discussion

Providing essential planned and elective care during an ongoing pandemic is challenging. Failing to restore these services may result in increased ‘non-COVID’ related morbidity and mortality as patients avoid hospital and healthcare contacts and delay important treatments because of fears and apprehensions about the risk of contracting COVID-19 in these settings [4, 12].

Our study shows that of the measures introduced by health care providers to facilitate safe planned care in the setting of COVID-19, patient testing and the separation of patient cohorts and pathways are perceived by patients to be most important.

It is interesting that patients identified patient testing as an important contributor to their safety but considered staff testing to be less important. Similarly, patients did not consider the extended 14-day pre-procedure isolation that they were required to undertake before their procedure to be important for their safety. This corresponds with anecdotal reports of variable compliance with recommended pre-procedure isolation.

Clearly, our study recruited patients who had self-selected to undergo elective treatment during the COVID-19 pandemic despite the level of fear and apprehension that many patients reported. It is to be expected that there is a cohort of patients who have declined such treatment and likely are not reassured by the measures put in place to make elective care safe in the setting of COVID-19. Our study could not include these patients but, their views and concerns will also be important in planning the meaningful reorganization of elective services.

The term ‘elective’ can sometimes suggest that care is in some way optional. In many cases this is not the case and elective care can be life changing and essential to patients in order to restore quality of life and to relieve pain and functional limitations [3, 8, 13]. Safely restarting elective services to allow important planned investigations, treatments and procedures to take place, even as we continue to manage the COVID-19 pandemic, is a priority. Restoring patient and public confidence in the safety of treatment pathways will be key. Patient priorities and perceptions of safety are important considerations.

## Conclusion

Patient and public confidence will be key to the success of any plans to restore planned and elective care. Our study suggests that a dedicated and real-time programme for patient testing and the physical separation of patient pathways impact most on patient confidence.

## Data Availability

Anonymized datasets used and/or analysed not including identifiable patient information are available from the corresponding author on reasonable request.

## Abbreviations

COVID-19: Coronavirus disease 2019
COVID: Coronavirus disease
NHS: National Health Service
SARS-CoV-2: Severe acute respiratory syndrome coronavirus 2
